# Antithrombin lowering in hemophilia: a closer look at fitusiran

**DOI:** 10.1016/j.rpth.2023.100179

**Published:** 2023-05-16

**Authors:** Guy Young, Peter J. Lenting, Stacy E. Croteau, Beatrice Nolan, Alok Srivastava

**Affiliations:** 1 Hemostasis and Thrombosis Center, Cancer and Blood Diseases Institute, Children’s Hospital Los Angeles, University of Southern California Keck School of Medicine, Los Angeles, California, USA; 2 Laboratory for Hemostasis, Inflammation and Thrombosis, Unité Mixed de Recherche, Institut National de la Santé et de la Recherche Médicale, Université Paris-Saclay, Le Kremlin-Bicêtre; 3 Boston Hemophilia Center, Boston Children’s Hospital, Boston, Massachusetts, USA; 4 Children’s Health Ireland at Crumlin, Dublin, Ireland; 5 Department of Haematology, Christian Medical College, Vellore, India

**Keywords:** antithrombin, fitusiran, hemophilia, small interfering RNA, thrombin

## Abstract

Thrombin is a key enzyme in the maintenance of normal hemostatic function and is the central product of an interconnected set of simultaneously occurring cellular and proteolytic events. Antithrombin (AT) is a natural anticoagulant that downregulates different components of the clotting process, particularly thrombin generation. In good health, well-regulated hemostasis is the result of a balance between procoagulant and anticoagulant elements. Cumulative understanding of the regulation of thrombin generation and its central role in hemostasis and bleeding disorders has led to the clinical development of therapeutic strategies that aim to rebalance hemostasis in individuals with hemophilia and other coagulation factor deficiencies to improve bleeding phenotype. The aim of this review is to discuss the rationale for AT lowering in individuals with hemophilia, with a focus on fitusiran, its mechanism of action, and its potential as a prophylactic therapy for individuals with hemophilia A or B, with or without inhibitors. Fitusiran is an investigational small, interfering RNA therapeutic that targets and lowers AT. It is currently in phase III clinical trials and results have shown its potential to increase thrombin generation, leading to enhanced hemostasis and improved quality of life while reducing the overall treatment burden.

## Essentials

• Hemostasis is achieved when sufficient thrombin is generated to form a stable fibrin clot.
• Fitusiran is a nonfactor therapy that lowers antithrombin (AT) to enhance thrombin generation.
• Fitusiran dosing now targets AT levels of 15% to 35% aiming to enhance its benefit-risk profile.
• Risk of thrombosis is greater with AT levels <10%, even in individuals with hemophilia.

## Graphical abstract

**Figure:**
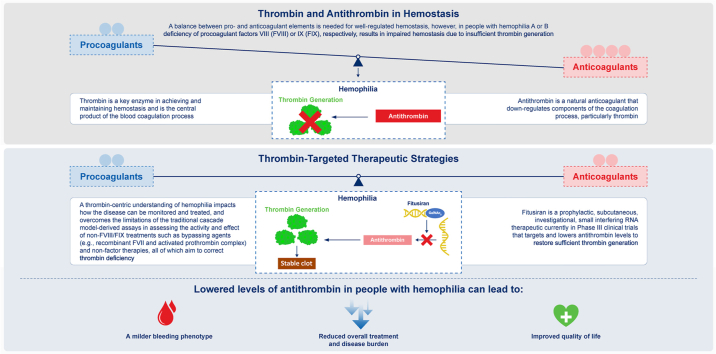

## 1 Introduction

**Figure 1:**
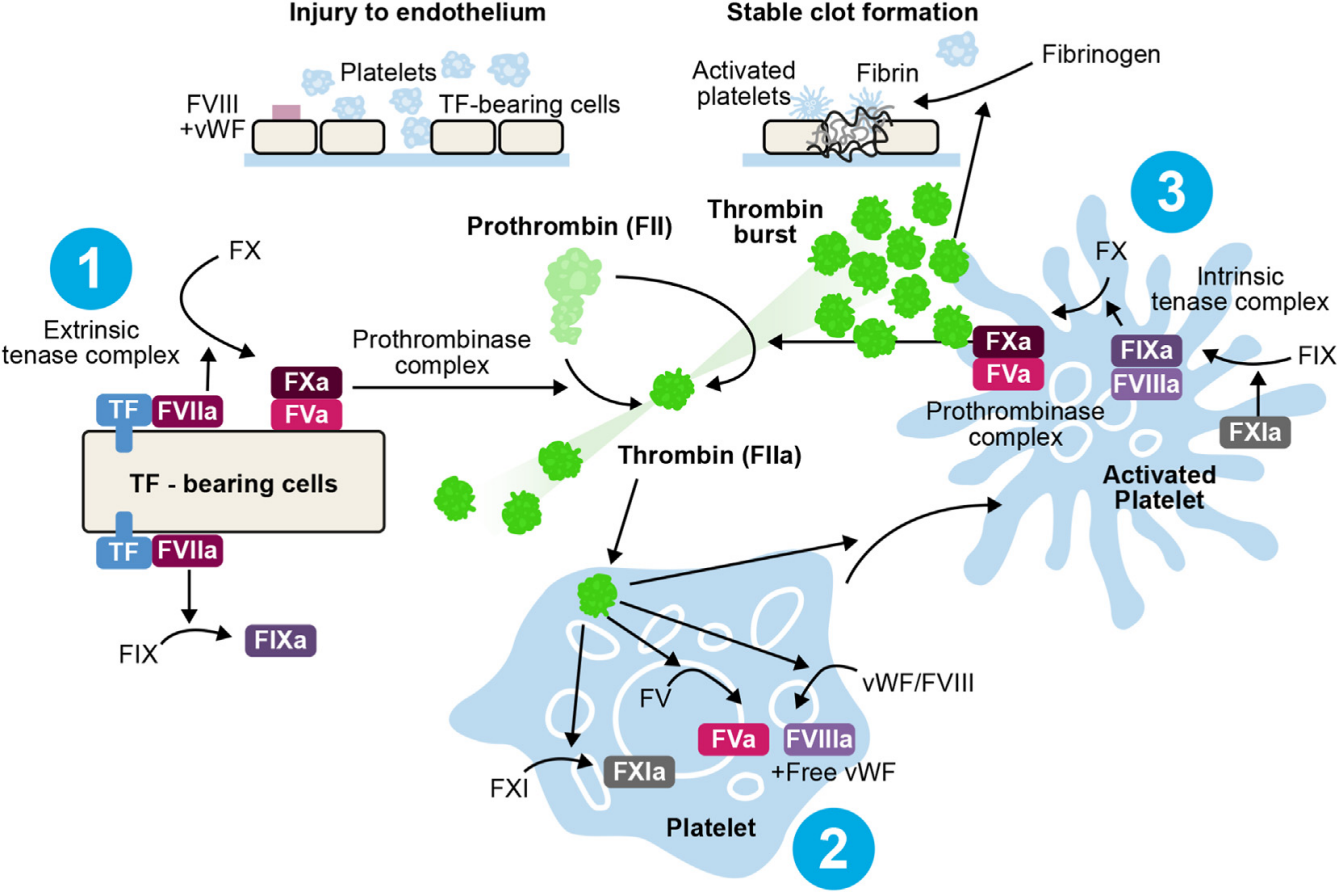
The cell-based model of initiation (1), amplification (2), and propagation (3) leading to coagulation. FV, factor V; FVa, activated factor V; FVIIa, activated factor VII; FVII; factor VII; FVIII, factor VIII; FIX, factor IX; FIXa, activated factor IX; TF, tissue factor; vWF, von Willebrand factor.

The hemostatic system is designed to respond to vascular injury, aiming to reduce blood loss and maintain hemostasis and the integrity of blood circulation, thereby preventing life-threatening hemorrhage [[1], [2], [3]]. In 2001, Hoffman and Monroe proposed the thrombin-centric, cell-based model of coagulation in which coagulation takes place on different cell surfaces in 3 phases (Figure 1). This cell-based model describes how coagulation is prevented from spreading throughout the vascular system and is confined to the site of injury. Additionally, the cell-based model allows a more detailed understanding of how hemostasis occurs *in vivo* compared with the canonical coagulation cascade model and can be used to help explain the pathophysiological mechanisms of certain coagulation disorders including hemophilia [4]. Despite differences in the detailed description of how coagulation functions, all models have in common that they culminate in the formation of thrombin as the key enzyme necessary for the formation of a fibrin network and stable clot [[5], [6], [7]].

**Figure 2:**
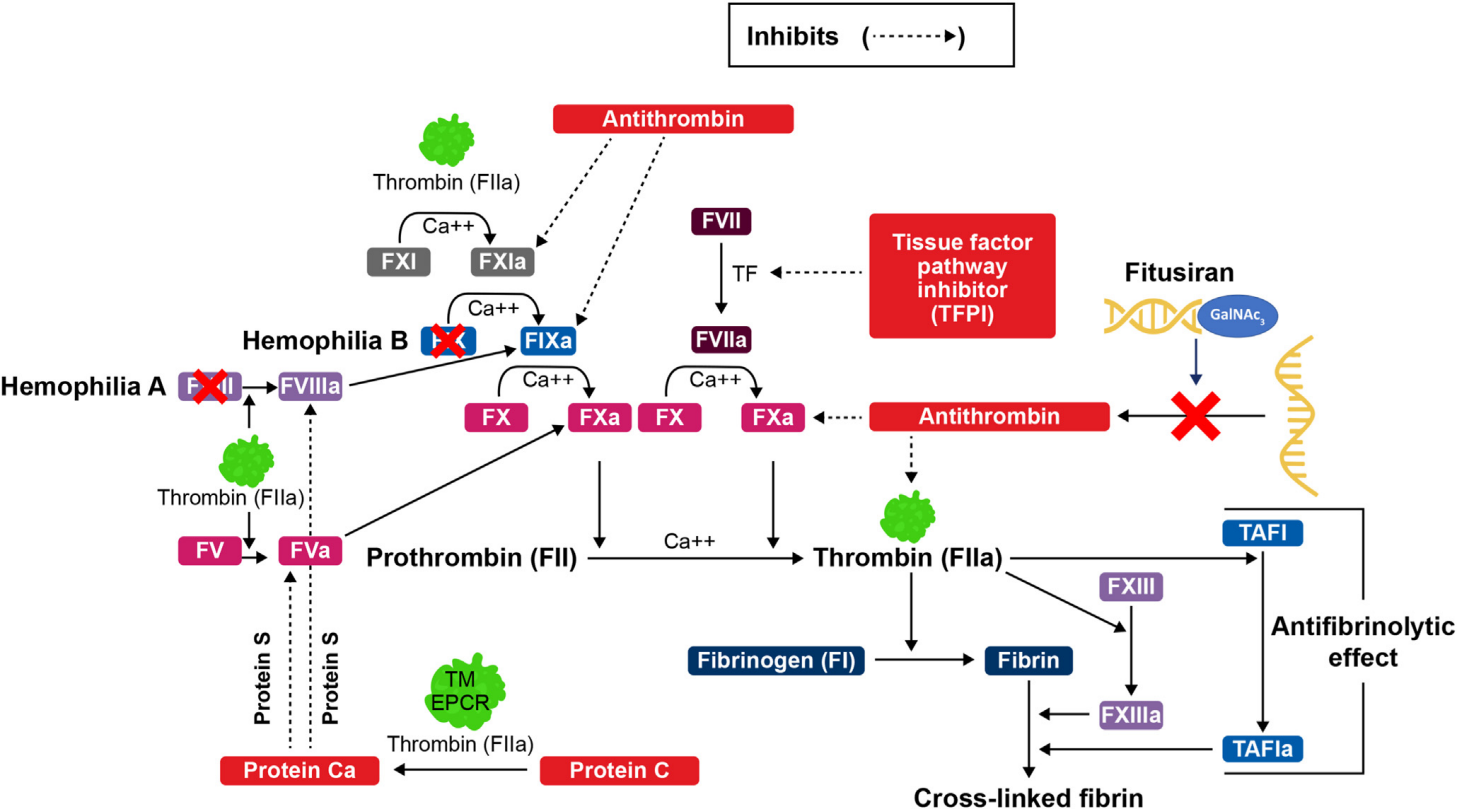
The role of thrombin and antithrombin in hemostasis, and the mechanism of action of fitusiran. EPCR, endothelial cell protein C receptor; FII, factor II; FIIa, activated factor II; FV, factor V; FVa, activated factor V; FVII, factor VII; FVIIa, activated factor VII; FVIII, factor VIII; FVIIIa, activated factor VIII; FIX, factor IX; FIXa, activated factor IX; FX, factor X; FXa, activated factor X; FXI, factor XI; FXIa, activated factor XI; FXIII, factor XIII; FXIIIa, activated factor XIII; Protein Ca, activated protein C; TAFI, thrombin activatable fibrinolysis inhibitor; TF, tissue factor; TM, thrombomodulin.

The regulation of thrombin formation is the result of a balance between procoagulant and anticoagulant proteins, and the absence of key proteins in this process may result in thrombotic or hemorrhagic complications [2]. For example, in individuals with hemophilia A or B, deficiency or dysfunction of factor (F)VIII (FVIII) or factor FIX, respectively, results in insufficient thrombin generation resulting in impaired hemostasis and uncontrolled or excessive bleeding, whereas antithrombin (AT) deficiency alone otherwise leads to thrombosis (Figure 2) [2].

To restore hemostasis in individuals with hemophilia the standard of care is prophylaxis, which aims to achieve no spontaneous bleeding [8]. Available treatment strategies rely on either replacing or mimicking the missing factors [9]. The major complication of clotting factor concentrate (CFC) therapy is the development of inhibitors, or neutralizing antibodies to replacement factor, with ∼30% of individuals with severe hemophilia A [10] and ∼10% of those with severe hemophilia B developing inhibitors [11].

In individuals with hemophilia and high-titer inhibitors, bypassing agents (BPAs) such as activated prothrombin complex concentrate and activated recombinant FVII (rFVIIa) can be used to treat acute bleeding events and as prophylaxis [12]. However, the use of BPAs has several limitations, including a short half-life, which means that they have limited efficacy as prophylactic agents used for the prevention of bleeding. They also require frequent infusions, increase the risk of thrombosis, and come at a high cost, which restricts access [[13], [14], [15], [16], [17]].

Nonfactor therapies are currently being used or investigated for prophylaxis in individuals with hemophilia and currently fall under 2 categories: therapies that mimic FVIIIa (substitution agents, ie, humanized anti-FIXa/FX bispecific antibodies such as emicizumab or investigational Mim8) and therapies that interfere with anticoagulant pathways (rebalancing agents; ie, AT-lowering, small interfering RNA therapy, such as fitusiran, anti-tissue factor pathway inhibitor antibodies such as concizumab or marstacimab, or therapies that target APC-dependent pathways) [[18], [19], [20]]. All of these therapies can be administered subcutaneously, most have long half-lives and necessitate, in general, infrequent administration, which may reduce treatment burden and increase the ability to deliver prophylaxis [19,21]. While these treatments are promising for prophylaxis, other hemostatic agents (CFC and BPAs) continue to be required for breakthrough bleeding or surgery [21].

The purpose of this review is to raise awareness of the rationale for the AT-lowering approach as a treatment strategy for people with hemophilia focusing on fitusiran, its mechanism of action, and its potential use as prophylactic treatment for individuals with hemophilia A or B, with or without inhibitors.

## 2 A Closer Look at AT Lowering

### 2.1 Hemophilia and AT deficiency

Hemophilia bleeding phenotype is defined by the clinical severity of bleeding as a balance of all hemostatic parameters, as well as the levels of the deficient or dysfunctional factors [22]. The severity of bleeding phenotype for individuals with hemophilia appears to correlate with thrombin generation, which is measured by global hemostatic assays [[23], [24], [25], [26]]. Part of this variation in thrombin generation and clinical phenotype can be explained by the variation in levels of anticoagulant proteins among individuals [27].

AT is the key anticoagulant enzyme of the hemostatic system (Figure 2). Heparin-activated AT inhibits coagulation by neutralizing thrombin and FXa and to a lesser extent FⅨa, FXIa, FXIIa, and other procoagulants [2,14,15]. Given its capacity to neutralize multiple targets, AT not only interferes with the generation of thrombin but also efficiently inhibits thrombin once generated [2]. Evidence suggests there is a modulation of bleeding tendency by factors in the anticoagulant and fibrinolytic systems [16].

AT deficiency was first described in 1965 [17]. and is associated with an increased risk for venous thrombosis as a result of reduced regulation of procoagulant proteins [28]. The coinheritance of prothrombotic traits in those with hemophilia, such as AT deficiency, has been found to be associated with a milder bleeding phenotype and an increased event-free bleeding survival rate [16,29]. Thus, reduced AT levels are hypothesized to improve thrombin generation and promote hemostasis, resulting in a potentially milder bleeding phenotype in individuals with hemophilia [16,29]. Antithrombin was therefore deemed an attractive, thrombin-target therapeutic strategy to explore for enhancing hemostasis in people with hemophilia [30].

### 2.2 Unique mechanism of action of fitusiran

In 1998, Fire and Mello published their landmark article, providing the first demonstration that RNA interference (RNAi) is triggered by double-stranded RNA and could repress the expression of a single gene [31]. These findings paved the way for the development of treatments, such as fitusiran, which is a subcutaneously administered, small, interfering RNA therapeutic agent that harnesses natural cellular RNAi mechanisms to cleave and degrade AT mRNA and reduce AT levels [32].

Small interfering RNA therapeutics are the most commonly used RNAi tools that harness the natural RNAi process. They consist of a synthetic RNA duplex designed to specifically target a particular mRNA for degradation. This prevents translation of the specific target mRNA, thus inhibiting protein synthesis [33].

AT, a natural anticoagulant with normal levels in the range of 80 to 120 IU/dL, is synthesized in the liver. Fitusiran is targeted to the liver by conjugation to N-acetylgalactosamine, a ligand for the asialoglycoprotein receptor located on hepatocytes [[32], [33], [34], [35]]. Fitusiran utilizes enhanced stabilization chemistry-N-acetylgalactosamine conjugate technology, which enables subcutaneous dosing, with increased potency and durability [34,36,37]. Fitusiran is not thought to be suitable for pediatric populations aged <1 year, as AT levels increase with age and do not reach adult values until about 6 months of age [38].

Pharmacokinetic analysis in clinical studies has shown that fitusiran has a short half-life in plasma (∼3-5 hours); however, lower AT levels persist for several months after discontinuation of fitusiran at all dose regimens tested, with a mean rate of AT recovery of 10% to 15% per month accompanied by a decrease in thrombin generation and an increase in bleeding events [32,39]. The median percent AT was found to increase to >60% after a 5-month period compared with the last measurement before dosing interruption [40].

The target pharmacodynamic effect of AT lowering has been shown to occur between 15 and 28 days after the administration of the first dose of fitusiran. After this onset period, it has been found that while there is interindividual variation in AT levels, there is minimal intraindividual variation in AT lowering, which potentially allows for more constant hemostatic protection among variable doses [32,39].

It has been proposed that reversal agents for nonfactor therapies may be useful, as the risk of thrombosis may potentially be increased when combining nonfactor therapies with other hemostatic agents to manage breakthrough bleeds. Currently, fitusiran is the only nonfactor therapy that has a specific reversal agent available in the form of recombinant or plasma-derived AT concentrates [41]. Evidence also demonstrates that decreased AT levels in plasma do not affect standard coagulation laboratory assays. This is important as it means that hemostasis and factor levels can easily be monitored in patients receiving fitusiran who may require CFC or BPAs to treat breakthrough bleeds and during some surgeries [42].

### 2.3 Preclinical proof of concept for fitusiran

Preclinical studies in FVIII-deficient mice with heterozygous AT deficiency showed that moderately reduced AT levels increased thrombin generation and decreased bleeding after tail clipping, suggesting that bleeding phenotypes can be modulated by the balance between procoagulant and anticoagulant proteins [43]. In addition to this, a second study demonstrated that when administered subcutaneously, fitusiran showed potent, dose-dependent, and durable reductions in AT levels in wild-type mice, mice with hemophilia A, and nonhuman primates with anti-FVIII inhibitors, resulting in improved thrombin generation [44]. Seghal et al. [44] used a saphenous vein bleeding model to investigate the *in vivo* efficacy of fitusiran in comparison with FVIII replacement. They observed that FVIII-deficient mice receiving 10 mg/kg fitusiran responded similarly to mice receiving 25-IU/kg FVIII concentrate. A more recent study, which used a Quantitative Systems Pharmacology model to explore hemostatic equivalency of AT lowering, indicated that in a virtual population with severe hemophilia A, targeted AT levels of 15% to 35% result in a peak thrombin levels similar to that associated with 20% to 40% of FVIII activity [45].

### 2.4 Clinical development of fitusiran

**Figure 3:**
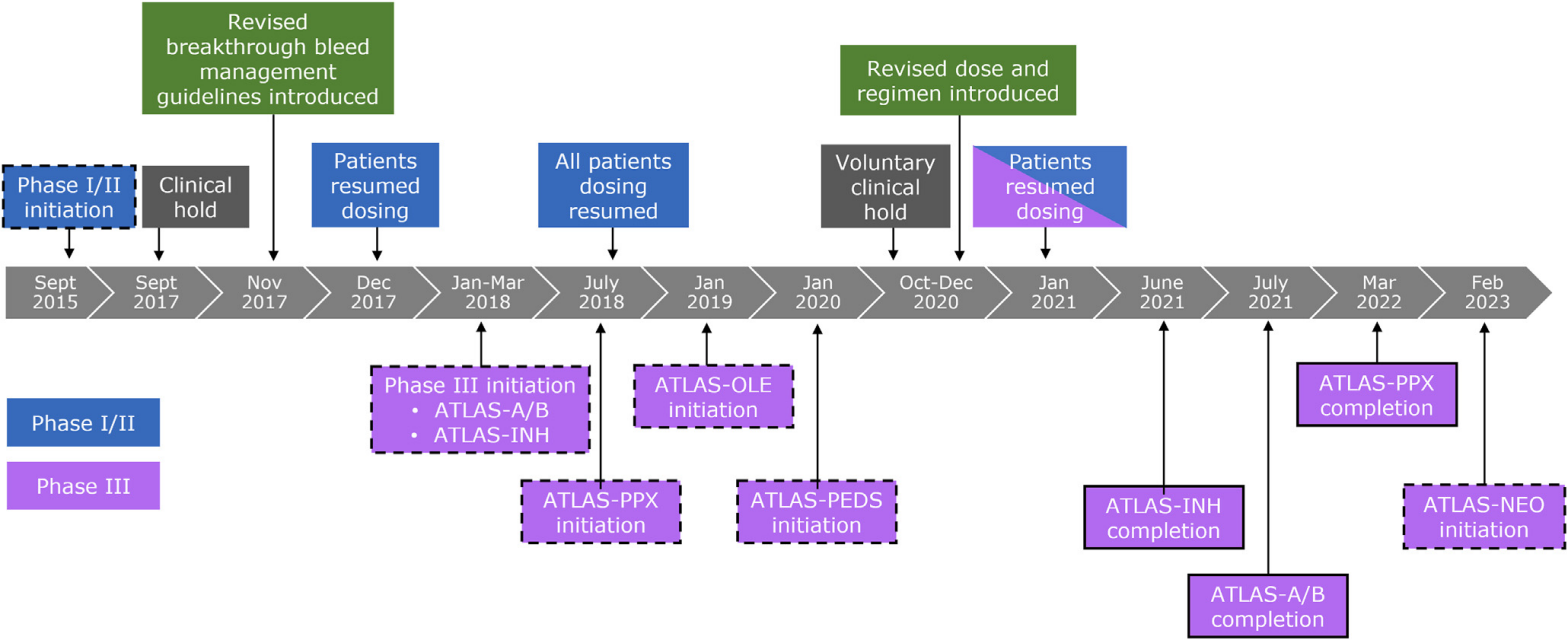
Key dates in the chronology of the fitusiran clinical development program. The black dashed lines indicate study initiation. The black solid lines indicate study completion.

As of February 2023, fitusiran is in phase III of clinical development for the treatment of individuals with hemophilia A or B, with or without inhibitors. Key dates in the chronology of the fitusiran clinical development program are presented in Figure 3. The clinical trial program consists of a completed 4-part phase I study (NCT02035605), an ongoing phase II open-label extension study (NCT02554773), 3 completed phase III studies (NCT03417102, NCT03417245, NCT03549871), one active-not recruiting phase III study (NCT03754790), and 2 actively-recruiting phase III studies (NCT03974113 and NCT05662319) (Table 1) [[46], [47], [48], [49], [50], [51], [52], [53]].

**Table 1:** Clinical program of fitusiran.

Study (NCT number)	Number and type of participants (N)	Dosing schedule	Key study endpoints	Status
Phase I, Part A [32] (NCT02035605)Timeframe: through day 56 (SAD phase)	*N* = 4 healthy participants	Fitusiran 0.03 mg/kg SC once	Primary:Safety and tolerabilitySecondary:PharmacokineticsPharmacodynamics	Completed
Phase I, Part B [32] (NCT02035605)Timeframe: through day 70 (MAD phase)	*N* = 12 people with moderate/severe hemophilia A/B with previous prophylaxis	Fitusiran 0.015, 0.045 or 0.075 mg/kg SC QW x 3		
Phase I, Part C [32] (NCT02035605)Timeframe: through day 112 (MD phase)a	*N* = 18 people with moderate/severe hemophilia A/B with previous prophylaxis^a^	Fitusiran 0.225, 0.45, 0.9 or 1.8 mg/kg or 80 mg SC QM x 3		
Phase I, Part D [39] (NCT02035605)Timeframe: through day 112 (MD phase in patients with inhibitors)	*N* = 17 people with moderate/severe hemophilia A/B, with inhibitors	Fitusiran 50 or 80 mg SC QM ×3		
Phase II open-label extension [46,55] (NCT02554773)	*N* = 34 people with moderate/severe hemophilia A/B, with or without inhibitors who tolerated fitusiran in Phase I (Parts B, C, and D)	Fitusiran 50 or 80 mg SC QM, then following the revised protocol as of December 2020	Primary:Long-term safety and tolerabilitySecondary:ABRAssessment of concomitantly administered FVIII, FIX, or BPA for bleeding episodesHRQoLPharmacokineticsPharmacodynamicsPlasma AT levelsPlasma thrombin generation	Active, not recruiting
Phase III ATLAS-A/B [51,57] (NCT03417245)	*N* = 120 people with severe hemophilia A/B without inhibitors previously treated on-demand	Fitusiran 80 mg SC QM prophylaxis (*n* = 80)On-demand factor concentrates (*n* = 40)Duration: 9 mo	Primary:ABRSecondary:Spontaneous ABRJoint ABRHRQoLTEAEs and SAEs	Completed
Phase III ATLAS-INH [50,56] (NCT03417102)	*N* = 57 people with severe hemophilia A/B with inhibitors previously treated on demand	Fitusiran 80 mg SC QM prophylaxis (*n* = 38)On-demand BPAs (*n* = 19)Duration: 9 mo	Primary:ABRSecondary:Spontaneous ABRJoint ABRHRQoLTEAEs and SAEs	Completed
Phase III ATLAS-PPX [49,58] (NCT03549871)	*N* = 80 people with severe hemophilia A/B previously receiving factor or BPA prophylaxis	Fitusiran 80 mg SC QM for 7 mo	Primary:ABRSecondary:Spontaneous ABRJoint ABRHRQoLTEAEs (19 mo [including up to 6 mo of AT follow-up])	Completed
Phase III ATLAS- open-label extension [48] (NCT03754790)	Actual enrollment: *N* = 355 people with hemophilia with or without inhibitory antibodies (FVIII, FIX)	Fitusiran 80 mg SC QM for 48 months then following the revised regimen	Primary:Safety and tolerabilitySecondary:ABRSpontaneous ABRJoint ABRHRQoL	Active, not recruiting
Phase III ATLAS-PEDS [47] (NCT03974113)	Estimated enrollment: *N* = 32 male pediatric participants (aged 1 to <12 y) with severe hemophilia A/B with inhibitors	Fitusiran SC at regular intervals as per study protocol	Primary:Plasma AT activity levels at the end of the efficacy period (approximately 160 wk)Secondary:AEs (160 wk)Fitusiran plasma concentrations (days 1 and 85)	Active, recruiting
Phase III ATLAS-NEO [53] (NCT05662319)	Estimated enrollment: *N* = 75 male adult and adolescent ≥12 y old with hemophilia A/B with or without inhibitory antibodies (FVIII, FIX)	In SoC period, on-demand or prophylactic treatment with CFCs or BPAs for 6 mo. In fitusiran treatment period, SC fitusiran prophylaxis Q2M or QM for 36 mo	Primary:ABR in the fitusiran efficacy periodSecondary:ABR while on fitusiran prophylaxis and ABR while on SoC prophylaxisABR while on fitusiran prophylaxis and ABR while on on-demand SoCSpontaneous ABR in the fitusiran efficacy period and the SoC periodJoint ABR in the fitusiran efficacy period and the SoC periodHaem-A-QoL in the fitusiran efficacy period and the SoC periodAE	Active, recruiting

ABR, annualized bleeding rate; AE, adverse event; AT, antithrombin; BPA, bypassing agent; CFC, clotting factor concentrate; FIX, factor IX; FVIII, factor VIII; FX, factor X; Haem-A-QoL, The Haemophilia Quality of Life Questionnaire for Adults; HRQoL, health-related quality of life; MAD, multiple-ascending dose; MD, multiple dose; QM, once-monthly; Q2M, once every other month; QW, once-weekly; SAD, single-ascending dose; SAE, serious adverse event; SoC, standard of care; TEAE, treatment emergent adverse event.

a Until AT activity returns to ≥80% of levels measured at screening.

To note, as of December 2020, protocol amendments were made affecting all ongoing studies in the fitusiran clinical development program to mitigate the risk of thrombosis with fitusiran. These changes are discussed in detail in section 2.6. The efficacy data reported here relates to data published prior to the protocol amendments, which affected the dose and regimen of fitusiran.

### 2.5 Reported efficacy and potential use of fitusiran prophylaxis in people with hemophilia

Fitusiran aims to rebalance hemostasis through AT lowering in individuals with hemophilia, irrespective of inhibitor status, leading to a sustained increase in thrombin generation and improved stable clot formation (Figure 2).

**Figure 4:**
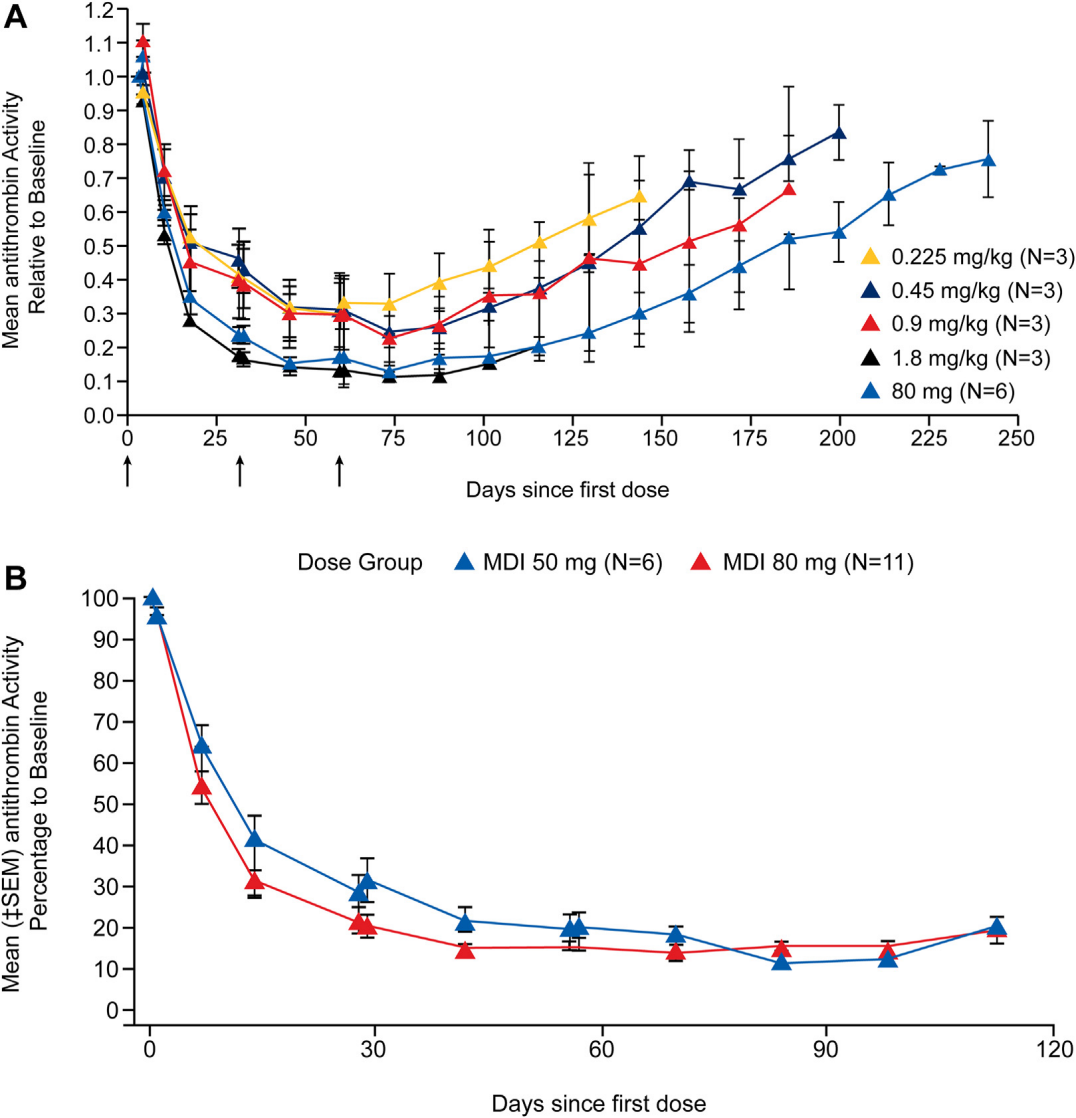
Effect of fitusiran on antithrombin activity in phase I study in people with hemophilia with inhibitors. (A) Phase I Part C. (B) Phase I Part D. (A) The arrows below the graph indicate the timing of the injections. The I bars represent standard errors, which were calculated only for cohorts with at least two participants. *Source:* Reproduced with permission from Pasi et al. [32]. (B) MDI, multiple doses with inhibitor; SEM, standard error of mean [39]. *Source:* Reproduced with permission from Pasi et al. [39].

In part C of the phase I fitusiran clinical trial, once-monthly s.c. administration of fitusiran demonstrated dose-dependent mean maximum lowering in AT levels by 70% to 89% from baseline and increased thrombin generation in participants with hemophilia A or B without inhibitors (Figure 4A) [32]. A *post hoc* exploratory analysis determined that monthly fitusiran dosing resulted in fewer bleeding episodes per month following treatment with fitusiran than before treatment [32]. Consistent with these results, in part D of the phase I trial, participants with hemophilia A or B with inhibitors, who received once-monthly administration of subcutaneous doses of fitusiran at 50 mg and 80 mg and were followed up for 112 days, experienced AT reductions from baseline of 82.0% and 87.4%, respectively, at nadir (Figure 4B). The reduction in AT activity was associated with increased thrombin generation [39]. Additionally, 64.7% of participants had no bleeds during the observation period (4 weeks after the first dose to 8 weeks after the last dose; mean, 69.4 days) with mean changes from baseline in the Haemophilia Quality of Life Questionnaire for Adults total and physical health domain scores, suggesting that there was a clinically meaningful improvement in quality of life compared with published thresholds; reductions of 10- and 7-units in the “Physical health” and “Total score” domains, respectively [39,54].

As of March 10, 2020, an interim analysis of the phase II open-label extension study showed that once-monthly fitusiran dosing achieved sustained AT lowering of ∼80%, resulting in peak thrombin levels approaching the lower range observed in healthy volunteers [55]. Exploratory *post hoc* analysis of bleeding events (follow-up of up to 4.7 years; median, 2.6 years) revealed a lower rate of bleeding episodes than before study enrollment. The median annualized bleeding rate (ABR) and the median annualized spontaneous bleeding rate were 0.84 and 0.34, respectively, for all participants (with or without inhibitors) during the observation period (the period following the 28-day onset period for fitusiran during which AT levels are expected to be within the target range) [55].

Analysis from phase III trial ATLAS-INH demonstrated that once-monthly administration of 80 mg fitusiran prophylaxis resulted in a significantly lower rate of bleeding events than with on-demand BPA treatment (median observed ABR [IQR], 0.0 [0.0-1.7] vs 16.8 [6.7-23.5]; *P* < .0001) among people with hemophilia A or B with inhibitors, with 65.8% of participants in the fitusiran arm with zero treated bleeding events [56]. Similarly, analysis from the ATLAS-A/B trial showed a significant reduction in bleeding events with fitusiran prophylaxis compared with on-demand CFC treatment (median observed ABR [IQR], 0.0 [0.0-3.4] vs 21.8 [8.4-4.1]; *P* < .0001) in participants with severe hemophilia A or B without inhibitors, with 50.6% of participants in the fitusiran arm with zero treated bleeding events [57]. A statistically significant improvement in physical health domain score with fitusiran vs. the on-demand arm was also observed in both studies indicating a meaningful improvement in health-related quality of life [56,57]. In addition, recent analysis from the ATLAS-PPX study has revealed that once-monthly administration of 80 mg fitusiran prophylaxis significantly reduced bleeding events compared with CFC/BPA prophylaxis (median observed ABR [IQR], 0.0 [0.0-2.3] vs 4.4 [2.2-10.9]) in participants with hemophilia A or B with or without inhibitors, with 63.1% of participants experiencing zero treated bleeds with fitusiran. This resulted in a significant improvement in health-related quality of life in the fitusiran arm compared with the CFC/BPA arm (least squares mean difference, −4.6 [95% CI, −7.6 to −1.5; *P* < .01]) [58].

### 2.6 Safety of fitusiran

In the phase I trial, 76% of the 25 participants with hemophilia who received fitusiran reported an adverse event (AE), with most of the events being mild to moderate in severity [32]. In part D of the phase I trial, no participants experienced serious drug-related AEs [39,59]. The most common drug-related AEs in the phase I study were injection-site pain and injection-site erythema. No AEs led to treatment discontinuation [32,39].

In phase I/II studies, a monthly fixed dose of fitusiran administered subcutaneously was evaluated in 25 participants with hemophilia A or B, with or without inhibitors. Following the release of phase II interim data, the study was placed on clinical hold on September 1, 2017, due to a fatal event of cerebral venous sinus thrombosis initially misdiagnosed as subarachnoid hemorrhage and accordingly treated with recommended doses of CFC per protocol [30,40]. Thrombotic risk mitigation strategies were implemented in November 2017, including revised breakthrough bleeding guidance informed by *in silico* modeling (Table 2), education of investigators and participants, and evaluation of symptoms suggestive of thrombosis [60]. Use of activated prothrombin complex concentrate and rFVIIa continued to be permitted for bleed treatment in participants receiving fitusiran but with a reduction in dose and frequency of infusions of hemostatic agents and avoidance of concomitant use of antifibrinolytics [30,60]. Following this, the study was restarted in December 2017 with regulatory authority approval (Figure 3).

**Table 2:** Revised breakthrough bleed management guidelines.

	Factor (F)VIII	FIX Standard half-life	FIX Extended half-life	APCC	rFVIIa
Recommended single dose	10 IU/kg	20 IU/kg	20 IU/kg	30 U/kg	≤45 μg/kg
Single dose should not exceed	20 IU/kg	30 IU/kg	30 IU/kg	50 U/kg	45 μg/kg
Repeat dosing	Must call clinical study center before second dose^a^; evaluation and treatment at clinical study center should be considered				Must call clinical study center before third dose^b^
	Should not repeat in <24 h		Should not repeat in <5-7 d	Should not repeat in <24 h	Should not repeat in <2 h
	For situations requiring higher doses, more frequent administration, or multiple repeated doses, discussion with study medical monitor and clinical advicer is recommended, and AT replacement should be considered.				
	Antifibrinolytics should not be used in combination with factor or BPA while on fitusiran.				

APCC, activated prothrombin complex concentrate; AT, antithrombin; BPA, bypassing agent; IU, international unit; rFVIIa, recombinant activated factor VII.

Table reproduced with permission from Pipe et al. [60].

a Should be seen at site within 48 to 72 hours if >2 doses are required.

b Should be seen at site within 48 to 72 hours if >3 doses are required.

**Figure 5:**
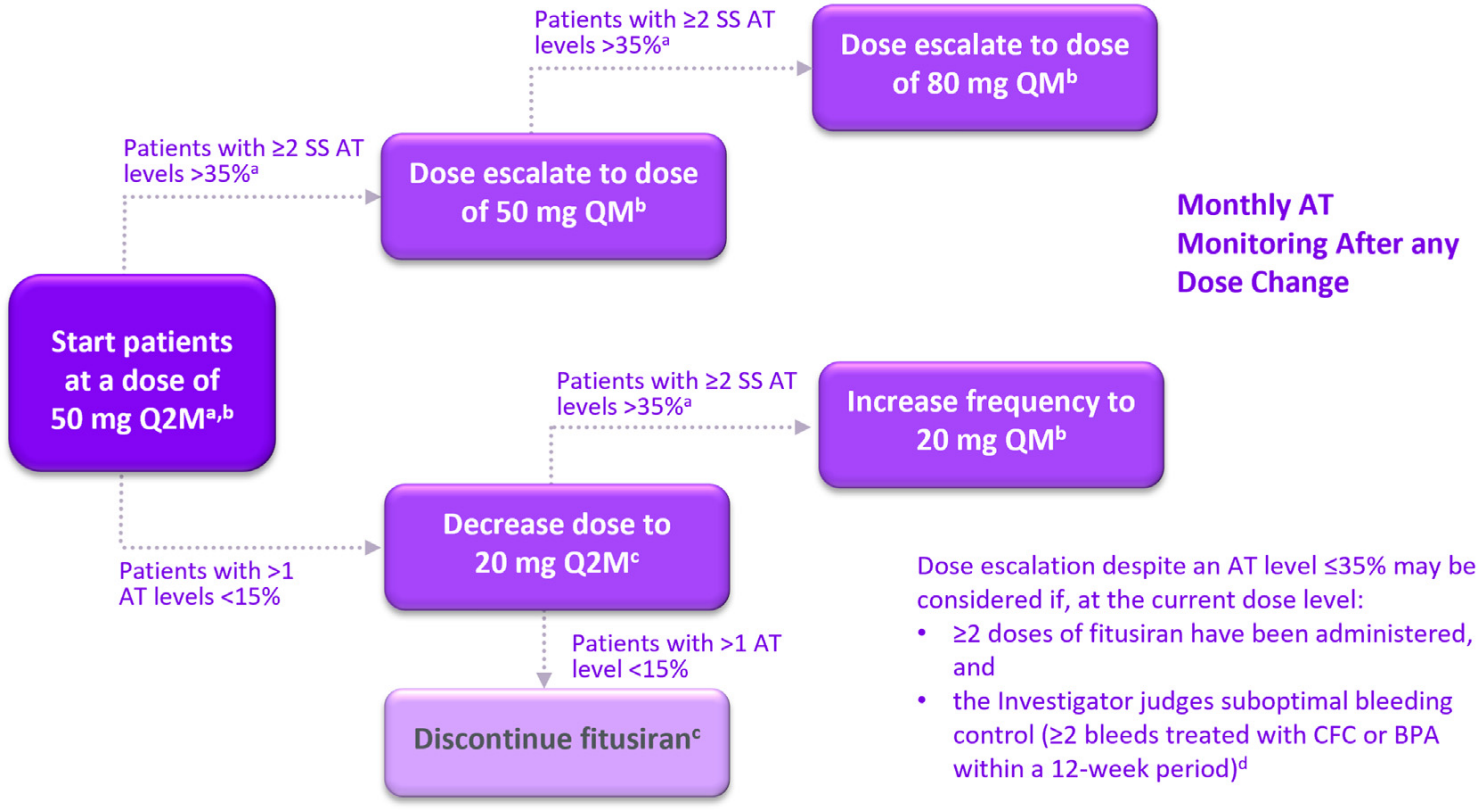
Fitusiran revised dose and dose regimen, targeting an antithrombin range from 15% to 35% [62]. The revised dose and dose regimen was introduced as of December 2020. BPA, bypassing agent; CFC, clotting factor concentrate; Q2M, every other month; SS, steady state. *Source:* Figure adapted with permission from Pipe et al. [62]. ^a^Participants are eligible for dose escalation if >4 doses of fitusiran have been administered at the current dose level, and they experienced >2 predose antithrombin (AT) activity levels >35% (as per central laboratory) after their second dose at the current dose, and fitusiran administration and AT activity assessments occurred as per schedule at the current dose level. ^b^Participants previously escalated to a dose of 20 mg every month (QM), 50 mg QM or 80 mg QM due to AT >35% who experience >1 AT activity level <15% within a 12 month period must either permanently discontinue fitusiran prophylaxis, or in consultation with the Study Medical Manager may have the option to be de-escalated to their prior dose level. ^c^Start of dosing after de-escalation from higher dose to occur only after centrally measured AT levels ≥22%. Participants receiving fitusiran at a dose of 20 mg every other month who experience ≥1 AT activity level <15% (as per central laboratory) within a 12-month period must permanently discontinue fitusiran treatment. ^d^Participants with QM dosing bleeding episodes during the first 8 weeks at the current dose level or every other month dosing bleeding episodes during the first 12 weeks at the current dose level will not be considered for this judgment.

On October 30, 2020, the sponsor voluntarily paused dosing in the ongoing fitusiran clinical studies to allow investigation of reports of nonfatal thrombotic events [61]. The investigation included analysis of reported thrombotic events as of October 2020, AT levels, and other available clinical data for all participants in the clinical development program (Table 3) [61]. In December 2020, fitusiran dosing resumed in the ongoing adult and adolescent clinical studies under amended protocols following approval by the regulatory authorities. The revised dose and regimen aimed at mitigating the thrombosis risk by modification of the fitusiran dose, dosing regimen, and target AT levels (Figure 5) [62]. The amended target AT levels were 15% to 35% rather than the previous goal of <10%. These changes are based on data suggesting an increased thrombotic risk in participants receiving fitusiran longitudinally with AT levels <10% [39,61]. Evaluation of the data indicated that in AT categories <10%, 10% to 20%, and >20%, the incident rate of thrombotic events per 100 patient-years was 5.91, 1.49, and 0, respectively [61]. Fitusiran has the potential for optimization of dose based on an individual’s response. The impact of revising the dose and regimen is currently under investigation.

**Table 3:** Evaluation of thrombotic events as of October 20, 2020, in the fitusiran clinical development program.^a^^,^^b^

Patient characteristics		Medical history/comments	AT category	Thrombotic event^c^
Age range (y)	Hemophilia subtype and inhibitor status			
30-40	Person with hemophilia A without inhibitor	Deep-vein thrombosis (not identified at enrollment; a study exclusion criterion), diabetes, obesity, HCV and tobacco use	<10%	Cerebrovascular accident
>60	Person with hemophilia A without inhibitor	Well-controlled HIV, HCV, and prostate cancer status-post radical prostatectomy with recent prostate-specific antigen within normal limits	<10%	Cerebral infarct
20-30	Person with hemophilia A with inhibitor	Suspected thrombosis involving a spinal injury	<10%	Spinal vascular disorder
20-30	Person with hemophilia B with inhibitor	Concomitant use of BPA (rFVIIa) in excess of the current bleed management guidelines in fitusiran clinical studies	10%-20%	Atrial thrombosis
20-30	Person with hemophilia A without inhibitor	Concomitant use of factor concentrate in excess of the current bleed management guidelines. Event was initially misdiagnosed and treated as a subarachnoid hemorrhage and resulted in a fatal outcome	10%-20%	Cerebral venous sinus thrombosis

AT, antithrombin; BPA, bypassing agent; HCV, hepatitis C.

Table adapted with permission from Negrier et al. [61].

a As of November 5, 2020, 259 participants have received at least 1 dose of fitusiran in the clinical development program, with an estimated total of 293 patient-years of exposure, excluding the data in phase I and pediatric studies.

b For all adult and adolescent patients exposed to at least 1 dose of fitusiran, the total patient-years for each of the 3 AT categories was calculated: <10%, 10% to 20%, and >20%. The patients with vascular thrombotic events were then included in the AT category representative of their level for the greatest amount of time during fitusiran exposure and an incident rate per 100 patient-years was derived.

c Adverse event data as of October 20, 2020.

Recent results from phase III trials ATLAS-INH, ATLAS-A/B, and ATLAS-PPX (NCT0341710, NCT03417245, and NCT03549871, respectively) demonstrated that reported treatment emergent AEs in the fitusiran prophylaxis arm were generally consistent with previously identified risks of fitusiran or what is anticipated in an adult and adolescent population with severe hemophilia A or B. The most common AEs reported in the fitusiran prophylaxis arm in the ATLAS-INH study, occurring in 12.2% of participants, were increased alanine aminotransferase, increased aspartate aminotransferase, upper abdominal pain, increased gamma-glutamyl transferase, headache, upper respiratory tract infection, arthralgia, increased blood alkaline phosphatase, and increased transaminases. Similarly, the most common AEs reported in the ATLAS-A/B study, occurring in 6.3% of participants, were increased alanine aminotransferase, upper respiratory tract infection, nasopharyngitis, abdominal pain, increased aspartate aminotransferase, cough, arthralgia, asthma, gastritis, and headache. In the ATLAS-PPX study, serious AEs were reported in 7.7% of participants treated with CFC/BPA prophylaxis and 13.4% of participants treated with fitusiran prophylaxis, with 2 participants (3.0%) experiencing suspected or confirmed thromboembolic events with fitusiran [[56], [57], [58],^63^].

## 3 Conclusions

Fitusiran is an investigational small interfering RNA therapeutic for subcutaneous prophylaxis in individuals with hemophilia A or B, irrespective of their inhibitor status, that has the potential to be transformative in hemophilia management through rebalancing of thrombin generation resulting in a milder bleeding phenotype, impacting the quality of life and reducing overall treatment burden. Ongoing clinical studies will provide further evidence on the efficacy and safety of fitusiran and its impact on patient-reported outcomes. It has been shown in preclinical *in vitro* and *in silico* studies that thrombin generation improves when fitusiran is added to plasma taken from patients with severe deficiency of FV, FVII, or FX [30]. Owing to its mechanism of action and thrombin-targeted approach, fitusiran therefore may be of use in other rare bleeding disorders that arise from insufficient thrombin generation, but further clinical studies are needed to confirm this hypothesis [30,44,64]. Overall, the evidence from clinical studies of fitusiran suggests that the benefits of the drug outweigh the risks [[55], [56], [57]]. and that fitusiran has the potential to change future clinical practice in hemophilia.

## Relationship Disclosure

G.Y. has received contracts for Sanofi clinical trials, consulting fees, and support from Sanofi for travel to attend meetings. P.J.L. has received grants or contracts from Sanofi and has given lectures for Sanofi. S.E.C. has received grants or contracts from Sanofi and has participated on an advisory board for Sanofi. B.N. has received contracts for 10.13039/100004339Sanofi clinical trials. A.S. has received grants or contracts from 10.13039/100004339Sanofi and has participated in advisory boards for 10.13039/100004339Sanofi.

## References

[1] Chaudhry R., Usama S.M., Babiker H.M. (2021).

[2] Negrier C., Shima M., Hoffman M. (2019). The central role of thrombin in bleeding disorders. Blood Rev.

[3] LaPelusa A., Dave H.D. (2022).

[4] Hoffman M., Monroe D.M. (2001). A cell-based model of hemostasis. Thromb Haemost.

[5] Macfarlane R.G. (1964). An enzyme cascade in the blood clotting mechanism, and its function as a biochemical amplifier. Nature.

[6] Davie E.W., Ratnoff O.D. (1964). Waterfall sequence for intrinsic blood clotting. Science.

[7] Hoffman M. (2003). A cell-based model of coagulation and the role of factor VIIa. Blood Rev.

[8] Srivastava A., Santagostino E., Dougall A., Kitchen S., Sutherland M., Pipe S.W. (2020). WFH guidelines for the management of hemophilia, 3rd edition. Haemophilia.

[9] Peyvandi F., Garagiola I., Young G. (2016). The past and future of haemophilia: diagnosis, treatments, and its complications. Lancet.

[10] Meeks S.L., Batsuli G. (2016). Hemophilia and inhibitors: current treatment options and potential new therapeutic approaches. Hematology Am Soc Hematol Educ Program.

[11] Male C., Andersson N.G., Rafowicz A., Liesner R., Kurnik K., Fischer K. (2021). Inhibitor incidence in an unselected cohort of previously untreated patients with severe haemophilia B: a PedNet study. Haematologica.

[12] Shapiro A.D., Mitchell I.S., Nasr S. (2018). The future of bypassing agents for hemophilia with inhibitors in the era of novel agents. J Thromb Haemost.

[13] Al Dieri R., Peyvandi F., Santagostino E., Giansily M., Mannucci P.M., Schved J.F. (2002). The thrombogram in rare inherited coagulation disorders: its relation to clinical bleeding. Thromb Haemost.

[14] Regnault V., Hemker H.C., Wahl D., Lecompte T. (2004). Phenotyping the haemostatic system by thrombography—potential for the estimation of thrombotic risk. Thromb Res.

[15] Butenas S., van ‘t Veer C., Mann K.G. (1999). “Normal” thrombin generation. Blood.

[16] Shetty S., Vora S., Kulkarni B., Mota L., Vijapurkar M., Quadros L. (2007). Contribution of natural anticoagulant and fibrinolytic factors in modulating the clinical severity of haemophilia patients. Br J Haematol.

[17] Egeberg O. (1965). Inherited antithrombin deficiency causing thrombophilia. Thromb Diath Haemorrh.

[18] Nogami K., Shima M. (2019). New therapies using nonfactor products for patients with hemophilia and inhibitors. Blood.

[19] Franchini M., Mannucci P.M. (2018). Non-factor replacement therapy for haemophilia: a current update. Blood Transfus.

[20] Lauritzen B., Bjelke M., Björkdahl O., Bloem E., Keane K., Kjalke M. (2022). A novel next-generation FVIIIa mimetic, Mim8, has a favorable safety profile and displays potent pharmacodynamic effects: results from safety studies in cynomolgus monkeys. J Thromb Haemost.

[21] Jimenez-Yuste V., Auerswald G., Benson G., Dolan G., Hermans C., Lambert T. (2021). Practical considerations for nonfactor-replacement therapies in the treatment of haemophilia with inhibitors. Haemophilia.

[22] Ibrahim U.A., Ahmed S.G. (2018). Determinants and modifiers of bleeding phenotypes in haemophilia-A: general and tropical perspectives. Egypt J Med Hum Genet.

[23] Brummel-Ziedins K.E., Whelihan M.F., Gissel M., Mann K.G., Rivard G.E. (2009). Thrombin generation and bleeding in haemophilia A. Haemophilia.

[24] Al Hawaj M.A., Martin E.J., Venitz J., Barrett J.C., Kuhn J.G., Nolte M.E. (2013). Monitoring rFVIII prophylaxis dosing using global haemostasis assays. Haemophilia.

[25] Dave R.G., Geevar T., Mammen J.J., Vijayan R., Mahasampath G., Nair S.C. (2021). Clinical utility of activated partial thromboplastin time clot waveform analysis and thrombin generation test in the evaluation of bleeding phenotype in hemophilia A. Indian J Pathol Microbiol.

[26] Jimenez-Yuste V., Auerswald G., Benson G., Lambert T., Morfini M., Remor E. (2014). Achieving and maintaining an optimal trough level for prophylaxis in haemophilia: the past, the present and the future. Blood Transfus.

[27] Jayandharan G.R., Srivastava A. (2008). The phenotypic heterogeneity of severe hemophilia. Semin Thromb Hemost.

[28] Lijfering W.M., Brouwer J.L., Veeger N.J., Bank I., Coppens M., Middeldorp S. (2009). Selective testing for thrombophilia in patients with first venous thrombosis: results from a retrospective family cohort study on absolute thrombotic risk for currently known thrombophilic defects in 2479 relatives. Blood.

[29] Ghosh K., Shetty S., Mohanty D. (2001). Milder clinical presentation of haemophilia A with severe deficiency of factor VIII as measured by one-stage assay. Haemophilia.

[30] Machin N., Ragni M.V. (2018). An investigational RNAi therapeutic targeting antithrombin for the treatment of hemophilia A and B. J Blood Med.

[31] Zamore P.D. (2006). RNA interference: big applause for silencing in Stockholm. Cell.

[32] Pasi K.J., Rangarajan S., Georgiev P., Mant T., Creagh M.D., Lissitchkov T. (2017). Targeting of antithrombin in hemophilia A or B with RNAi therapy. N Engl J Med.

[33] Hu B., Weng Y., Xia X.H., Liang X.J., Huang Y. (2019). Clinical advances of siRNA therapeutics. J Gene Med.

[34] Huang C.M., Kroll M.H., Ruddel M., Washburn R.G., Bennett J.E. (1988). An enzymatic method for 5-fluorocytosine. Clin Chem.

[35] Van Cott E.M., Orlando C., Moore G.W., Cooper P.C., Meijer P., Marlar R. (2020). Recommendations for clinical laboratory testing for antithrombin deficiency; communication from the SSC of the ISTH. Thromb Haemost.

[36] Foster D.J., Brown C.R., Shaikh S., Trapp C., Schlegel M.K., Qian K. (2018). Advanced siRNA designs further improve in vivo performance of GalNAc-siRNA conjugates. Mol Ther.

[37] Nair J.K., Willoughby J.L., Chan A., Charisse K., Alam M.R., Wang Q. (2014). Multivalent N-acetylgalactosamine-conjugated siRNA localizes in hepatocytes and elicits robust RNAi-mediated gene silencing. J Am Chem Soc.

[38] Catrine A., Nilsson T. (1975). Antithrombin in infancy and childhood. Acta Paediatr Scand.

[39] Pasi K.J., Lissitchkov T., Mamonov V., Mant T., Timofeeva M., Bagot C. (2021). Targeting of antithrombin in hemophilia A or B with investigational siRNA therapeutic fitusiran-Results of the phase 1 inhibitor cohort. J Thromb Haemost.

[40] Ragni M.V., Georgiev P., Creagh M.D., Lissitchkov T., Austin S.K., Hay C.R.M. (2018). The role of antithrombin lowering in patients with hemophilia: hemostatic control pre- and post-fitusiran dosing interruption. Blood.

[41] Arruda V.R., Doshi B.S., Samelson-Jones B.J. (2018). Emerging therapies for hemophilia: controversies and unanswered questions. F1000Res.

[42] Wang S., Kattula S., Ismail A., Leksa N., van Der Flier A., Salas J. (2020). Reducing Antithrombin in plasma to levels observed in fitusiran-treated patients does not interfere with coagulation assays. Blood.

[43] Bolliger D., Szlam F., Suzuki N., Matsushita T., Tanaka K.A. (2010). Heterozygous antithrombin deficiency improves in vivo haemostasis in factor VIII-deficient mice. Thromb Haemost.

[44] Sehgal A., Barros S., Ivanciu L., Cooley B., Qin J., Racie T. (2015). An RNAi therapeutic targeting antithrombin to rebalance the coagulation system and promote hemostasis in hemophilia. Nat Med.

[45] Kaddi C., Tao M., Leiser R., Salvador A., Kattula S., Bhagunde P. (2022). Development of a quantitative systems pharmacology model to explore hemostatic equivalency of antithrombin lowering. Blood.

[46] ClinicalTrials.gov (2022). An open-label extension study of an investigational drug, fitusiran, in patients with moderate or severe hemophilia A or B. https://clinicaltrials.gov/ct2/show/NCT02554773?term=NCT02554773&draw=2&rank=1.[Accessed.

[47] ClinicalTrials.gov (2022). Fitusiran prophylaxis in male pediatric subjects aged 1 to less than 12 years with hemophilia A or B (ATLAS-PEDS). https://clinicaltrials.gov/ct2/show/NCT03974113?term=NCT03974113&draw=2&rank=1.[Accessed.

[48] ClinicalTrials.gov (2022). Long-term safety and efficacy study of fitusiran in patients with hemophilia A or B, with or without inhibitory antibodies to factor VIII or IX (ATLAS-OLE). https://clinicaltrials.gov/ct2/show/NCT03754790?term=NCT03754790&draw=2&rank=1.[Accessed.

[49] ClinicalTrials.gov (2022). A study of fitusiran in severe hemophilia A and B patients previously receiving factor or bypassing agent prophylaxis (ATLAS-PPX). https://clinicaltrials.gov/ct2/show/NCT03549871?term=NCT03549871&draw=2&rank=1.

[50] ClinicalTrials.gov (2022). A study of fitusiran (ALN-AT3SC) in severe hemophilia A and B patients with inhibitors (ATLAS-INH). https://clinicaltrials.gov/ct2/show/NCT03417102?term=NCT03417102&draw=2&rank=1.[Accessed.

[51] ClinicalTrials.gov (2022). A study of fitusiran (ALN-AT3SC) in severe hemophilia A and B patients without inhibitors. https://clinicaltrials.gov/ct2/show/NCT03417245?term=NCT03417245&draw=2&rank=1.[Accessed.

[52] ClinicalTrials.gov (2022). A phase 1 study of an investigational drug, ALN-AT3SC, in healthy volunteers and hemophilia A or B patients. https://clinicaltrials.gov/ct2/show/NCT02035605?term=NCT02035605&draw=2&rank=1.[Accessed.

[53] ClinicalTrials.gov (2023). A study to test a medicine (fitusiran) injected under the skin for preventing bleeding episodes in male adolescent or adult participants with severe hemophilia (ATLAS-NEO). https://clinicaltrials.gov/ct2/show/NCT05662319.

[54] Wyrwich K.W., Krishnan S., Poon J.L., Auguste P., von Maltzahn R., Yu R. (2015). Interpreting important health-related quality of life change using the Haem-A-QoL. Haemophilia.

[55] Pipe S.W., Pasi J., Lissitchkov T., Ragni M.V., Negrier C., Yu Q. (2020).

[56] Young G., Srivastava A., Kavakli K., Ross C., Sathar J., Tran H. (2021). Efficacy and safety of fitusiran prophylaxis, an siRNA therapeutic, in a multicenter phase 3 study (ATLAS-INH) in people with hemophilia A or B, with inhibitors (PwHI). Blood.

[57] Srivastava A., Rangarajan S., Kavakli K., Klamroth R., Kenet G., Khoo L. (2021). Fitusiran, an investigational siRNA therapeutic targeting antithrombin for the treatment of hemophilia: first results from a phase 3 study to evaluate efficacy and safety in people with hemophilia a or B without inhibitors (ATLAS-A/B). Blood.

[58] Kenet G., Nolan B., Zulfikar B., Antmen B., Kampmann P., Matsushita T. (2022). A phase 3 study (ATLAS-PPX) to evaluate efficacy and safety of fitusiran, an siRNA therapeutic, in people with haemophilia A or B who have switched from prior factor or bypassing agent prophylaxis. Res Pract Thromb Hemost.

[59] Piñeiro-Carrero V.M., Piñeiro E.O. (2004). Liver. Pediatrics.

[60] Pipe S., Ragni M.V., Négrier C., Yu Q., Bajwa N., Caminis J. (2019). Fitusiran, an RNAi therapeutic targeting antithrombin to restore hemostatic balance in patients with hemophilia A or B with or without inhibitors: management of acute bleeding events. Blood.

[61] Negrier C., Pasi K.J., Ragni M., Pipe S.W., Cue Y., Bhagunde P. (2021).

[62] Pipe S.W., Srivastava A., Klamroth R., Kenet G., Tran H., Fetita L. Fitusiran, an investigational siRNA therapeutic targeting antithrombin: analysis of antithrombin levels and thrombin generation from a phase 3 study in people with hemophilia A or B without inhibitors. https://abstracts.isth.org/abstract/fitusiran-an-investigational-sirna-therapeutic-targeting-antithrombin-analysis-of-antithrombin-levels-and-thrombin-generation-from-a-phase-3-study-in-people-with-hemophilia-a-or-b-without-inhibitors/.

[63] Sanofi Data from two phase 3 studies demonstrating fitusiran significantly reduced bleeds in people with hemophilia A or B, with or without inhibitors, were featured at ASH’s plenary and late-breaking sessions [press release]. https://www.sanofi.com/en/media-room/press-releases/2021/2021-12-14-14-00-00-2351761.

[64] Sridharan G., Tsour S., Liu J., Qian K., Goel V., Huang S. (2017). In silico modeling of the coagulation cascade and thrombin generation: simulating antithrombin (AT) lowering in hemophilia and rare bleeding disorders (RBDs). Blood.

